# Supplementary material for: PLOS Genetics 2014 Reviewer Thank You
Source: PLoS Genet. 2015 Feb 27;11(2):e1005070. doi: 10.1371/journal.pgen.1005070 (PMC4344251; doi:10.1371/journal.pgen.1005070)
Supplement: S1 Reviewer List — (PDF) [file pgen.1005070.s001.pdf]

PLOS | GENETICS

PLOS Genetics would like to thank all those who reviewed on behalf of the journal in 2014:

Stuart Aaronson
Alejandro Aballay
Tarek Abbas
Allison Abbott
Jessica Abbott
Abdel Abdellaoui
Zalfa Abdel-Malek
Gonçalo Abecasis
Asa Abeliovich
Susan Abmayr
Roger Abounader
John Abrams
Usha Acharya
Annita Achilleos
Martin Ackermann
Josephine Adams
Michael Adams
David Adams
Ian Adams
Peter Adams
Karen Adelman
Paul Adler
Markus Affolter
Alessandra Agresti
Andrés Aguilera
Nadav Ahituv
Kami Ahmad
Yashi Ahmed
Shawn Ahmed
Brahim Aissani
Elias Aizenman
Joshua Akey
Marie-Andree Akimenko
Mirit Aladjem
Eric Alani
Victor Albert
Simon Alberti
David Albertini
Arthur Alberts
Urs Albrecht
Joy Alcedo
Mattias Alenius
Juan Alfonzo
Nazif Alic
Ravi Allada
Douglas Allan
Thaddeus Allen
Robin Allshire
Juan Alonso
Berta Alsina
James Alspaugh
Andre Altmann
Carlos Alvarez
José Álvarez-Castro
James Amatruda
David Amberg
Michael Amling
Gustav Ammerer
Christopher Amos
Simon Anders
Svend Andersen
James Anderson
Tim Anderson
Carl Anderson
Dan Andersson
Leif Andersson
Peter Andolfatto
Ole Andreassen
Rose Andrew
Deborah Andrew
Joe Angel
Montserrat Anguera
Robert Anholt
Mireille Ansaldi
Adam Antebi
Hana Antonicka
Agostinho Antunes
Fugaku Aoki
Ruslan Aphasizhev
Peter Aplan
Suneel Apte
Charles Aquadro
Johan Åqvist
Luís Aragón
Eli Arama
Alexei Aravin
Benoit Arcangioli
Jon Arch
Detlev Arendt
Manuel Ares
Ralph Arlinghaus
Judith Armitage
David Arnosti
David Arnot
Carlo Artieri
Kristin Artinger
Masako Asahina
Hosseinali Asgharian
Ruth Ashery-Padan
Kaveh Ashrafi
Stella Aslibekyan
Stefan Astrom
John Atkins
Yurii Aulchenko
Steven N. Austad
Adam Auton

Leon Avery
Michael Axtell
David Aylor
Hans Peter Bächinger
Helmut Bäumlein
Isabel Bäurle
Astrid Böhne
G. Valentin Börner
Willy Baarends
Paul Babitzke
Erika Bach
Ingolf Bach
Francois Bachand
Jeff Bachant
Charles Baer
Melanie Bahlo
Yong-Sun Bahn
Feng-Yan Bai
Adam Bailis
Duncan Baird
Ruchi Bajpai
Keith Baker
Suzanne Baker
Jeroen Bakkers
Gabor Balazsi
Richard Baldock
Robert Baloh
Leah Band
Utpal Banerjee
Vikas Bansal
Fernando Baquero
Pavel Baranov
Thomas Baranski
Daniel Barbash
Jordi Barbe
Scott Barbee
Lisa Barcellos
Allison Bardin
Barbara Bardoni
James Bardwell
Naama Barkai
Alice Barkan
Maria Barna
Scott Barolo
Martin Baron
Maureen Barr
Yves Barral
Frédéric Barras
François-Xavier Barre
Antoni Barrientos
Gregory Barsh
Nick Barton
Michelle Barton
Kerstin Bartscherer
Jeremy Baskin
Konrad Basler
Munira Basrai
Hank Bass
Joseph Bass
Alexander Bassuk
Deepak Bastia
Boris Bastian
Joseph Bateman
Gillian Bates
David Bates
Brendan Battersby
Michele Battle
Jacques Batut
L. Baugh
Ralf Baumeister
Mary Baylies
Cynthia Beall
Wendy Beane
Terri Beaty
Tommaso Beccari
Philip Becraft
Attila Becskei
Nigel Beebe
Nicola Beer
Christian Beetz
Gerrit Begemann
Chase Beisel
Greg Beitel
Robert Belas
William Belden
Sandra Beleza
Marlene Belfort
Tildon Belgard
Douglas Bell
Jordana Bell
Hugo Bellen
Deborah Bell-Pedersen
John Belmont
Mark Belmonte
Boris Belotserkovskii
Jennifer Below
William Bendena
Arnold Bendich
Monsef Benkirane
Richard Bennett
Tom Bennett
Malcolm Bennett
Eric Bennett
Jörn Bennewitz
Andrew Benson
Eugene Berezikov
Howard Berg
Andrew Bergen
Frederic Berger
Dave Berger

Alan Bergland
Helmut Bergler
David Bergstrom
Judith Berman
John Bermingham
Paolo Bernardi
Thomas Bernhardt
Harris Bernstein
Alexandre Berr
Matthew Berriman
Claire Bertet
Olivier Berton
Lars Bertram
Alison Bertuch
Florence Besse
Jean Louis Bessereau
Joerg Betschinger
Marco Betti
Jill Bettinger
Regina Betz
Bruce Beutler
Andreas Beyer
Needhi Bhalla
Kapil Bharti
Manzoor Bhat
Sukesh Bhaumik
Peter Bickel
Sharon Bickel
Wendy Bickmore
Jason Bielas
Mariann Bienz
Laura Bierut
Stefano Biffo
Sue Biggins
Abigail Bigham
Margherita Bignami
Elaine Bignell
Oliver Billker
Brad Binder
Emanuele Biondi
James Birchler
Carmen Birchmeier
C. William Birky, Jr.
Ewan Birney
Douglas Bishop
Alexander Bishop
Eric Bittman
Stefan Björklund
Maria Björkqvist
Brian Black
Stephen Blacklow
Benjamin Blackman
Seth Blackshaw
Craig Blackstone
T. Keith Blackwell
Jenefer Blackwell
Patrick Blader
Seth Blair
Luis Blanco
Giovanni Blandino
William Blaner
Robert Blassberg
Justin Blau
Miguel Blazquez
John Blenis
Marnie Blewitt
Kerry Bloom
Cinnamon Bloss
Justin Blumenstiel
Frederic Boccard
Michael Boddy
Thomas Boehm
Michael Boehnke
Paolo Boffetta
Dan Bogenhagen
Magdelana Boguta
Didier Boichard
Ewelina Bolcun-Filas
Blaise Boles
Michael Bolker
Tobias Bollenbach
Dan Bolon
Kirsten Bomblies
Vera Bonardi
Ulla Bonas
Nancy Bonini
Joshua Bonkowsky
Robert Bonomo
Adam Book
Charles Boone
Vesna Boraska
Guntram Borck
Christelle Borel
Justin Borevitz
Tilman Borggrefe
Douglas Bornemann
Maria Bortolini
Elena Bosch
Thomas Bosch
Giovanni Bosco
Yohan Bossé
Lionello Bossi
Vladimir Botchkarev
Philippe Boucher
Jean-Yves Bouet
Simon Boulton
Kostas Bourtzis
Michael Boutros
Klaas Bouwmeester
Mondher Bouzayen

Paola Bovolenta
Margot Bowen
Bruce Bowerman
Josephine Bowles
Toby Bradshaw
Jason Bragg
Axel Brakhage
Marc Bramkamp
Thomas Brand
Federica Brandizzi
Yaniv Brandvain
Susan Branford
Robert Braun
David Braun
Gerhard Braus
Sarah Bray
Linda Breeden
Rachel Brem
Erhard Bremer
Paul Brennan
Julius Brennecke
Anthony Bretscher
Joshua Brickman
Miguel Brieño-Enriquez
Michael Briggs
Julie Brill
Steven Brill
James Briscoe
Normand Brisson
Steven Britt
James Broach
Peter Brodersen
Steven Brody
Heather Broihier
Marianne Bronner
Samantha Brooks
Brian Brooks
Phillip Brooks
Seth Brooks
David Brow
Susan Brown
Steven Brown
Grant Brown
Matthew Brown
Christopher Brown
Myles Brown
Carolyn Brown
Patrick Brown
Pamela Brown
Lynette Brownfield
Sharon Browning
Zabrina Brumme
Anne Brunet
Michael Brunner
Damian Brunner
Joseph Bruton
David Bryant
John Buchan
Gregor Bucher
Nicolas Buchon
Martin Buck
Wolfgang Buckel
Jane Buckner
Vivian Budnik
Yelena Budovskaya
Marc Buehler
Juan Bueren
Alex Buerkle
Alfonso Buil
Davide Bulgarelli
Martha Bulyk
Samuel Bunting
Stephen Buratowski
Robert Burgess
Harold Burgess
Molly Burke
Lori Burrows
Vincent Burrus
Graham Burton
William Bush
Kathryn Bushley
Allan Buskirk
Victor Busov
Dolores Busso
Ana Busturia
Michael Buszczak
J. Butler
Roger Butlin
Mark Buttner
Peter Byers
Ivalu Cacho
Ken Cadigan
Qiuyin Cai
Rodrigo Calado
John Calarco
Keith Caldecott
Kim Caldwell
Andrea Calixto
Patrick Callaerts
Sean Callahan
Edmundo Calva
Gerard Campbell
Elizabeth Campbell
Nathalie Campo
Sophie Candille
Christine Canman
Rafael Cantera
Carlos Canto
Xiaofeng Cao
Andrew Capaldi

Blanche Capel
Aurélien Capitan
Anthony J. Capobianco
Maria Cardenas
Valerio Carelli
Vincent Carey
Karen Carleton
Ross Carlson
Geert Carmeliet
Maria Carmo-Fonseca
Miguel Carneiro
Tom Carney
Antony Carr
Laura Carrel
Sebastien Carreno
Ana carrera
Jorge Casal
Claudia Casalongue
Jean-Laurent Casanova
Fernando Casares
Nicholas Casewell
Tamara Caspary
Diogo Castro
Sara Cathey
Gianpiero Cavalleri
Douglas Cavener
Cristina Cebrian
Mathieu Cellier
Sebastián Chávez
Andrei Chabes
Trinad Chakraborty
Aravinda Chakravarti
Douglas Chalker
Ronald Chalmers
Stormy Chamberlain
Severine Chambeyron
Yingguang Frank Chan
David Chan
Danny Chan
Raymond Chan
Perng Kuang Chang
Yun Chang
Hsiao-Han Chang
Caren Chang
Sandy Chang
Stephen Chanock
Jocelyn Chapman
Jean-Philippe Charles
Deborah Charlesworth
Brian Charlesworth
Dan Chase
Daniel Chasman
Keith Chater
Dhruba Chattoraj
Claire Chazaud
Michael Cheetham
Dahua Chen
Xin Chen
Z. Jeffrey Chen
Phang-Lang Chen
Lin Chen
Zheng-Yi Chen
David Chen
Wei-Min Chen
Chen Chen
Taiping Chen
Riyan Cheng
Shiyuan Cheng
Heung-Chin Cheng
Alice Chen-Plotkin
Yury Chernoff
Elissa Chesler
Zachary Cheviron
Jen-Tsan Chi
Ann-Shyn Chiang
Delphine Chinchilla
Ian Chin-Sang
Joanna Chiu
Giltsu Choi
Jung Kyoon Choi
Kyunghee Choi
King Chow
Beat Christen
Lane Christenson
Lionel Christiaen
Vincent Christoffels
Charleen Chu
Mon-Li Chu
Chengcai Chu
Gordon Chua
Sumeet Chugh
Ung-il Chung
Edward Chuong
Cheng-Ming Chuong
Karlene Cimprich
Rafal Ciosk
Elizabeth Cirulli
Andrew Clark
Scott Clark
Duncan Clarke
David Clayton
Christine Clayton
Ondine Cleaver
Stephan Clemens
Sharlee Climer
Joachim Clos
Beatrice Cobucci-Ponzano
Bryan Coburn
Bradley Coe
Paula Cohen

Laurie Cohen
Paul Cohen
Barak Cohen
Ronald Cohn
Francesca Cole
Benjamin Cole
Douglas Cole
Martine Collart
Philippe Collas
Jeff Coller
Jean-Francois Collet
Nansi Colley
Nigel Collier
Justine Collier
James Collins
Sheila Collins
Vincent Colot
Luca Comai
David Comas
Josep Comeron
Ciarán Condon
Tim Connallon
Karen Conneely
Barbara Conradt
Andrea Contestabile
Silvestro Conticello
Mark Cookson
Joseph Coolon
Jan Cools
Kathleen Cooney
Graham Coop
Geoffrey Cooper
Vaughn Cooper
Tim Cooper
Chester Cooper
Endymion Cooper
Thomas Cooper
William Copeland
Neal Copeland
Shelley Copley
Victor Corces
Richard Cordaux
Jeffry Corden
Dolores Corella
Omar Cornejo
Pierre Cornelis
Robert Cornell
Davide Corona
Carl Correll
Adrian Cortes
David Cortez
Marco Cosentino Lagomarsino
Chris Cotsapas
Fergus Couch
Sarah Coulthurst
Deborah Court
Juan Pablo Couso
Mark Cowley
Michael Cox
Nancy Cox
Timothy Cox
Nancy Craig
Erin Cram
Dana Crawford
Nigel Crawford
Frans Cremers
Bernard Crespi
W. Cress
Steve Crews
Daniel Croll
Philip Crosier
Jay Cross
Merlin Crossley
Sean Crosson
Gray Crouse
Michele Crozatier
Carlos Cruchaga
Gage Crump
Kyle Cunningham
Christina Cuomo
L. Adrienne Cupples
James Curley
Asher Cutter
Jack da Silva
Anupama Dahanukar
Stephen Daiger
Lisa Dailey
Colin Dale
Tamas Dalmay
Alex Dammermann
Weiwei Dang
Olga Danilevskaya
Michael Dapp
Jennifer Darnell
Seth Darst
Andrew Darwin
Jeremy Dasen
Brigitte Dauwalder
George Davey Smith
Patrice David
Jean-Philippe David
Lior David
R. Kelly Dawe
Dean Dawson
David Dawson
Paul de Bakker
Marc De Block
Piet de Boer
Mario de Bono
Valerie de Crécy-Lagard

Dirk-Jan de Koning
Pilar De la Rua
Nicholas De Lay
Gustavo de los Campos
Miguel de Pedro
Ive De Smet
Lieven De Veylder
Claudio De Virgilio
J. Arjan G. M. de Visser
Ann Dean
Antony Dean
Dennis Dean
Margaret DeAngelis
Seth DeBolt
Jason DeBruyne
Georges Dedoussis
Jaquelin DeFaveri
Michael DeGiorgio
Jacob Degner
Abbas Dehghan
Benjamin Dekel
Olivier Delaneau
Mary Delany
Clelia De-la-Peña
Franck Delaunay
Joy Delhanty
Christos Delidakis
Markus Delling
Alison DeLong
Panos Deloukas
Florence Demenais
Fabio Demontis
Anneke den Hollander
Erick Denamur
Benjamin Deneen
Christophe d'Enfert
Wu-Min Deng
Han-Xiang Deng
David Denlinger
Jonathan Dennis
Arshad Desai
Darrell Desveaux
Andrew Dewan
Javier Di Noia
Dion Kai Dickman
Thomas Dickmeis
Xavier Didelot
Giorgio Dieci
William Diehl
Paul Dijkwel
Joseph Dillard
Andrew Dillin
Niall Dillon
F. Jeffrey Dilworth
Shou-Wei Ding
Mei Ding
Jonathan Dinman
José Dinneny
Jose Dinneny
Daniela Dinulescu
Marc Dionne
Luisa DiPietro
James DiRenzo
Jocelyne DiRuggiero
Christine Disteche
Dan Dixon
Ray Dixon
Ron Do
Ulrich Dobrindt
Paul Doetsch
Aidan Doherty
Liam Dolan
Anne Donaldson
Xinnian Dong
Aiwu Dong
Nicole Donofrio
Erik Dopman
Robert Dorit
Charles Dorman
Dale Dorsett
Simon Dove
Frank Doyle
Sean Doyle
Cord Drögemüller
Jeremy Draghi
Thomas Drake
Gregory Dressler
Jean-Michel Drezen
Ines Drinnenberg
Alexander Drohat
Jacques Drouin
David Drubin
Iain Drummond
Dongsheng Duan
David Dubnau
Denis Duboule
Edward Dubrovsky
Frank Dudbridge
Joel Dudley
Aimee Dudley
Martin Duennwald
Janet Duerr
Michael Duffy
Beth Dumont
Ian Duncan
Christine Dunham
Malcolm Dunlop
Ian Dunn
Alison Dunning
Julie Dunning Hotopp

Gary Dunny
William Dunphy
Laurent Duret
Jonathan Dworkin
Michael Dyall-Smith
Fred Dyda
Kelly Dyer
Dan Dykhuizen
Brian Dynlacht
Walter Eanes
Johann Eberhart
Francis Ebling
Kristin Eckert
Christian Eckmann
Mitch Eddy
Edward Eddy
Isaac Edery
Bruce Edgar
Owain Edwards
Todd Edwards
Gerald Edwards
Boris Egger
Jean-Marc Egly
Måns Ehrenberg
Ian Ehrenreich
Melanie Ehrlich
Patrick Eichenberger
Evan Eichler
Eli Eisenberg
David Eisenmann
Karl Ekwall
Knust Elisabeth
Hannah Elliott
Peter Ellis
Nathan Ellis
Ronald Ellis
Steven Ellis
Roberto Elosua
Bert Ely
James Emerson
Patrick Emery
Ben Emery
Kazuo Emoto
Wolfgang Enard
JoAnne Engebrecht
Philipp Engel
Astrid Engel
Barbara Engelhardt
David Engelke
Anton Enright
Jose Antonio Enriquez
John Eppig
Scott Erdman
Albert Erives
Adrian Erlebacher
Jason Ernst
Klaus Ersfeld
Elhan Ersoz
Nilufer Ertekin-Taner
Yuval Eshed
Eleazar Eskin
Jeffrey Essner
Charles Ettensohn
Todd Evans
David Evans
Michael Evgen'ev
Jonathan Ewbank
Adam Eyre-Walker
Marie-Anne Félix
Fabio Facchinelli
Benjamin Fairfax
Damien Faivre
Jiri Fajkus
Maria Falkenberg
Hua-Ying Fan
Jinggui Fang
Qing Fang
Christian Fankhauser
Philip Farabaugh
Peggy Farnham
Diane Fatkin
Victor Faundez
Jeffrey Fawcett
David Fay
Justin Fay
David Featherstone
Michael Federle
Edward Feil
Andrew Feinberg
Wenyi Feng
Bing-Jian Feng
Michael Ferdig
Javier Fernandez-Martinez
Dominique Ferrandon
Elisabetta Ferretti
David Fidock
Stanley Fields
Dmitry Filatov
Guillaume Filion
Ruth Finkelstein
E. Jean Finnegan
Anthony Firulli
Robert Fischer
Woodward Fischer
Janice Fischer
Simon Fisher
Elizabeth Fisher
Lila Fishman
J. Ross Fitzgerald
Ben Fitzpatrick

David Fitzpatrick
Klas Flardh
Thomas Flatt
Ursula Fleig
Timothy Florin
Uta Flucke
Timothée Flutre
Brent Fogel
Elizabeth Fontes
James Ford
Chris Ford
Jiri Forejt
Pau Formosa-Jordan
Susan Forsburg
Mark Fortini
Kevin Foster
Fabienne Foufelle
Douglas Fowler
Edward Fox
Don Fox
Paul Francois
Olivier Francois
Alison Frand
C. Andrew Frank
Lude Franke
W. Anthony Frankino
Hunter Fraser
Timothy Frayling
Merete Fredholm
Michael Freitag
John French
Catherine Freudenreich
Erwin Frey
Marc Friedlander
Alan Friedman
Nir Friedman
Thorsten Friedrich
Jiri Friml
Menachem Fromer
Bryan Fry
Horacio Frydman
Judith Frydman
Michaela Frye
Xiang-Dong Fu
Robert Fuchs
Elaine Fuchs
Isabelle Fudal
Joachim Fuellekrug
Tatsuo Fukagawa
Robert Furbank
Denis Furling
Takahisa Furukawa
Anthony Futerman
John Fyfe
H. Ulrich Göringer
Boris Görke
Huldrych Günthard
Davide Gabellini
Daniel Gaffney
Phillip Gage
Dominique Gagliardi
Niels Galjart
Michael Galko
Kimberly Gallagher
Jennifer Gallagher
Imed Gallouzi
Eric Gamazon
Susheng Gan
Serge Gangloff
Maureen Gannon
Ziyue Gao
Guangxia Gao
Feng Gao
Tatiana García-Muse
Henri-Jean Garchon
L. Rene Garcia
Antonio Garcia de Herreros
Jose Garcia-Perez
Donald Gardiner
Katheleen Gardiner
Richard Gardner
David Garfield
David Garfinkel
Katherine Garman
Craig Garner
Victoriano Garre
Gian Garriga
Daniel Garrigan
Nanibaa' Garrison
Paul Garrity
Danielle Garsin
Marc Gartenberg
Charles Gasser
David Gatfield
Luke Gaughan
Kyle Gaulton
Sergey Gavrilets
Rasheed Gbadegesin
Kai Ge
Niels Gehring
Mary Gehring
Peter Geigenberger
Erika Geisbrecht
Johannes Geiselmann
David Geiser
Vincent Geli
Pierre Genevaux
Pavel Georgiev
André Gerber
Justin Gerke

Greg Germino
Jennifer Gerton
Jason Gertz
Daniel Geschwind
Jason Gestwicki
Pamela Geyer
Daniel Gianola
Greg Gibson
Christian Gieger
Clement Gilbert
David Gilbert
Lilach Gilboa
Matthew Gill
Thomas Gillingwater
Eric Gilson
Thomas Gingeras
Mark Ginsberg
Stephen Ginsberg
Matthew Giorgianni
Santhosh Girirajan
Aaron Gitler
Tom Glaser
Philippe Glaser
Christopher Glass
Jane Glazebrook
Michael Glickman
Alison Goate
Mike Goddard
John Godwin
Wolfram Goessling
David Goldgar
Lynn Goldin
Gustavo Goldman
Kent Golic
Mark Gomelsky
Mário Gomes-Pereira
Jose Luis Gomez-Skarmeta
Zachariah Gompert
Pierre Gonczy
Thomas Gonda
Venugopala Reddy Gonehal
Zhizhong Gong
Josefa González
Cristina Gonzalez-Estevez
Bruce Goode
John Goodier
Michael Goodisman
Andrew Goodman
Sofie Goormachtig
Isabel Gordo
Fred Gorelick
Michael Gorin
Olga Gorlova
Myriam Gorospe
Susan Gottesman
Kathleen Gould
Benjamin Gourion
Aska Goverse
J Gowrishankar
Sebastian Grönke
Ed Grabczyk
Gregory Grabowski
David Grainger
Beata Grallert
Sander Granneman
Chris Grant
Murray Grant
David Grattan
Peter Graumann
Dan Graur
Brenton Graveley
Jennifer Graves
Lucas Gray
Elizabeth Grayhack
Laura Greaves
Jeremy Green
Pamela Green
William Greenleaf
Ralph Greenspan
Celia Greenwood
Peter Gregersen
Thomas Gregor
Simon Gregory
Stephen Gregory
Sushma-Nagaraja Grellscheid
Shiv Grewal
Joachim Griesenbeck
Courtney Griffin
Malachi Griffith
Joel Griffitts
Andrew Grimson
Alla Grishok
Joanna Groden
Martien Groenen
Elisabeth Grohmann
Natalia Gromak
Leif Groop
Philippe Gros
Carol Gross
Joshua Gross
Jeffrey Gross
Rita Gross-Hardt
Enzo Grossi
Alan Grossman
Frank Grosveld
Andy Groves
Stephan Gruber
Jan Gruber
Jeffrey Gruen
Elin Grundberg

Pawel Grzechnik
Zhenglong Gu
Yongtao Guan
Johann Gudjonsson
Parry Guilford
Deborah Gumucio
Shermali Gunawardena
Teresa Gunn
Hongwei Guo
Yan Guo
Wei Guo
Deepti Gurdasani
Claes Gustafsson
Crisanto Gutierrez
Jose Gutierrez-Marcos
Monica Höfte
Stefan Höning
Eric Haag
Wolfgang Haak
Brian Haarer
Steven Haase
Bianca Haase
James Haber
Jason Hackney
Zena Hadjivasiliou
Daryl Hadsell
Udo Haecker
Gordon Hager
Paul Hagerman
Nobuko Hagiwara
Matthew Hahn
David Haig
Christopher Haiman
Alex Hajnal
Anders Hakansson
Sandra Hake
Sarah Hake
Regine Hakenbeck
Thanos Halazonetis
Georg Halder
Mario Halic
Jennifer Hall
David Hall
Sarah Hall
Martin Hallberg
Ronald G. Haller
Adrian Halme
Dennis Halterman
Tina Hambuch
Richard Hamelin
Christian Hammann
Matthias Hammerschmidt
Peter Hammond
Kim Hammond-Kosack
Leendert W. Hamoen
Iqbal Hamza
Junhai Han
Buhm Han
Ze-Guang Han
Dana Hancock
Mary Ann Handel
Jacob Hanna
Anthony Hannan
Lochmuller Hanns
Olivier Hanotte
Malene Hansen
John Hansen
Yu Hao
Rudolf Happle
Nicholas Harberd
Ross Hardison
Wolf-Dietrich Hardt
John Hardy
Richard Harland
Michael Harms
Dag Harmsen
Alison Harrill
Steven Harris
Reuben Harris
Matthew Harris
Peter Harris
Rasika Harshey
Matthew Hartfield
Richard Harvey
Caroline Harwood
Bassem Hassan
Mary Hatten
Theodora Hatziioannou
R. Scott Hawley
Shigeo Hayashi
Ken Haynes
Nick Hayward
Yuehui He
Jiang He
Housheng He
Denis Headon
Ulrike Heberlein
Katja Hebestreit
Angela Heck
David Heckel
Olaf Heidenreich
Yael Heifetz
Matthias Heinig
Erilynn Heinrichsen
Nathaniel Heintz
Carl-Philipp Heisenberg
Marcus Heisler
Jiri Hejnar
Yrjo Helariutta
Charlotte Helfrich-Forster

Gib Hemani
Myriam Hemberger
Ian Henderson
Steven Henikoff
Brenna Henn
Lars Hennig
Yves Henry
Kathryn Hentges
Joachim Hermisson
Ryan Hernandez
Alan Herr
David Herrin
Richard Herrmann
Ruth Hershberg
Anat Herskovits
Ronna Hertzano
Doris Herzlinger
Rex Hess
Evelyne Heyer
Holger Heyn
Michael Hickerson
Mark Hickman
Alicia Hidalgo
P. Robin Hiesinger
James Higgins
N. Higgins
Robert Hill
William Hill
Chris Hill
Gary Hime
Justin Hines
Patricio Hinrichsen
Jay Hinton
Deborah Hinton
Yasushi Hiraoka
Toru Hirota
Gideon Hirschfield
Christophe Hitte
Chris Hittinger
Robin Hobbs
Oliver Hobert
Asger Hobolth
Mark Hochstrasser
Andreas Hochwagen
Jan Hoeijmakers
Noah Hoffman
Eric Hoffman
Eva Hoffmann
Deborah Hogan
Paul Hohenlohe
Stefan Hohmann
Alexander Hoischen
James Holaska
Michelle Holland
Linda Holland
Nancy Hollingsworth
Joanna Holloway
Todd Holmes
Scott Holmes
Frank Holstege
Jeffrey Holt
Dave Hoon
Thorsten Hoppe
David Horn
Julia Horsfield
Valerie Horsley
William Horton
Tamas Horvath
Steve Horvath
Steven Hou
Corinne Houart
Eugene Houseman
Douglas Houston
Riekelt Houtkooper
Rosalind Howes
Bryan Howie
Jason Howitt
Hsu-Liang Hsieh
Ao-Lin Hsu
Fuqu Hu
Xiaoyu Hu
Patrick Hu
Jian Hua
Yingqun Huang
Suyun Huang
Jirong Huang
Taosheng Huang
Gang Huang
Jane Hubbard
Armin Huber
Damien Hudson
Jim Hughes
Kelly Hughes
Robert Hughes
Scott Hultgren
Tim Humphrey
Arthur Hunt
Neil Hunter
Craig Hunter
Peter Hurlin
Antonio Hurtado
Wieland Huttner
Jean-René Huynh
Michael Hynes
Michael Ibba
Oxana Ibraghimov-Beskrovnaya
Hidenori Ichijo
Youssef Idaghdour
Alexander Idnurm
Taisen Iguchi

Mark Iles
Robert Illingworth
Hae Kyung Im
Yuzuru Imai
Takato Imaizumi
Maneesha Inamdar
Nicholas Ingolia
Ituro Inoue
Masayori Inouye
Michael Inouye
Isabelle Iost
M. Kathryn Iovine
Grzegorz Ira
Hiroaki Ishikawa
Shalev Itzkovitz
Zoltan Ivics
Vishwanath Iyer
Elisa Izaurralde
Zsuzsanna Izsvák
Dean Jackson
F. Rob Jackson
Ian Jackson
Alain Jacquier
Johannes Jaeger
Hamed Jafar-Nejad
Andrew Jaffe
Mukesh Jain
Dagmara Jakimowicz
Mattias Jakobsson
Guilhem Janbon
Sharon Jansa
Gert Jansen
Verena Jantsch
Christian Janzen
Andrew Jarman
Heinrich Jasper
William Jeffery
Barbara Jennings
Jeffrey Jensen
Choongwon Jeong
Jukka Jernvall
Rolf Jessberger
Jianhang Jia
Songtao Jia
Yulin Jia
Ning Jiang
Jin Jiang
Yuling Jiao
Chris Jiggins
Rafael Jimenez
Peng Jin
Marek Jindra
Sue Jinks-Robertson
Frank Johannes
Hanna Johannesson
Kristen Johansen
Erik Johansson
Mina John
Welkin Johnson
Timothy Johnson
Kenneth Johnson
Mark Johnson
Jane Johnson
Louise Johnson
Norman Johnson
Andrew Johnson
Jean-Stephane Joly
Felicity Jones
David Jones
Peter Jones
Thomas Jongens
Marcel Jonkman
Catherine Jopling
Philip Jordan
Mathieu Joron
Emma Josefsson
Pradeep Joshi
Luke Jostins
Raja Jothi
Alexandra Joyner
Gabor Juhasz
Peter Juo
Ulrich Kück
Edor Kabashi
Sebastian Kadener
David Kadosh
Farid Kadyrov
Matt Kaeberlein
Klaus Kaestner
Jonathan Kagan
Jon Kaguni
Daniel Kalderon
Rohinton Kamakaka
Zachary Kaminsky
Ritsu Kamiya
Sophien Kamoun
Harm Kampinga
Masahiko Kanamori
Hyun Min Kang
Artur Kania
Patricia Kannouche
Patrick Kanold
Stavroula Kanoni
Aimee Kao
Craig Kaplan
Tarun Kapoor
Katrin Karbstein
Elinor Karlsson
Gary Karpen
Elizabeth Karr

Kathleen Karrer
David Kass
Naoyudi Kataoka
Sekar Kathiresan
Vaishali Katju
Aris Katzourakis
Paul Kaufman
Jim Kaufman
Liisa Kauppi
Yawara Kawano
Barbara Kazmierczak
Jennifer Kearney
Nancy Kedersha
Scott Keeney
Peter Keightley
Kenneth Keiler
Alon Keinan
Erin Kelleher
Mark Keller
Charles Keller
Beat Keller
Matthew Keller
Joanna Kelley
William Kelley
Robert Kelly
John Kelly
Morgan Kelly
Karl Kelsey
Gavin Kelsey
Carly Kenkel
Scott Kennedy
Batsheva Kerem
Andrew Kern
Johannes Kettunen
Philipp Khaitovich
Javed Khan
Sharik Khan
Peter Kharchenko
Jeff Kidd
Benjamin Kidder
Amy Kiernan
Abraham Kierszenbaum
Adam Kiezun
Amy Kiger
Kazu Kikuchi
Kiyoshi Kikuchi
Michael Kilberg
Patricia Kiley
John Kilmartin
J. Julie Kim
John Kim
Dennis Kim
Yuseob Kim
Nayun Kim
Judith Kimble
Charles Kimmel
Sarah Kimmins
Tohru Kimura
Tetsu Kinoshita
John Kirby
Laura Kirkman
Mark Kirkpatrick
David Kirkpatrick
Leif Kirsebom
Matias Kirst
Katsumi Kitagawa
Jan Kitajewski
Jun Kitano
Robert Kittel
Rick Kittles
Karin Klauke
Nancy Kleckner
Kenneth Kleene
Hannah Klein
Dirk-Jan Kleinjan
Daniel Klessig
Daniel Kliebenstein
Martin Klingenspor
Martin Klingler
Jochen Klumpp
Arne Klungland
Antti Knaapila
Paul Knoepfler
David Knowles
Karen Knudsen
Tatsuya Kobayashi
Takehiko Kobayashi
Michael Kobor
Lutz Kockel
Robert Kofler
Gou-Young Koh
Kyunghee Koh
Annegret Kohler
Hisato Kondoh
Alexey Kondrashov
Hongzhi Kong
Tomohiro Kono
Genevieve Konopka
Bon-Kyoung Koo
Eugene Koonin
Maarten Koornneef
Frank Kooy
Jeffrey Kopp
Jan Korbel
Kerry Kornfeld
Benoit Kornmann
Abraham Korol
Hendrik Korswagen
Arthur Korte
Sergei Kosakovsky Pond

Christian Kosan
Haruhiko Koseki
Carolin Kosiol
Sawa Kostin
Noora Kotaja
Zsofia Kote-Jarai
Irina Kovtun
Stephen Kowalczykowski
Leslie Kozak
Anita Kozyrskyj
James Kramer
Helmut Kramer
Ksenia Krasileva
Michael Krause
Rachel Kraut
Peter Krawitz
Jordan Kreidberg
Martin Kreitman
Karl Kremling
Dieter Kressler
Doris Kretzschmar
Skirmantas Kriaucionis
Robin Krimm
Beth Krizek
Guido Kroemer
Lee Kroos
Arthur Kruckeberg
Leonid Kruglyak
Karsten Kruse
Sergey Kryazhimskiy
Patrick Krysan
Karl Kuchler
Allison Kuipers
Rob Kulathinal
Anuj Kumar
Rajiv Kumar
Ashish Kumar
Justin Kumar
Shoen Kume
Martin Kupiec
Hiroshi Kurahashi
Deborah Kurrasch
Zoltán Kutalik
Ulrike Kutay
Chris Kuzawa
Andrei Kuzminov
Kristen Kwan
Pui-Yan Kwok
Michael Kyba
Charalambos Kyriacou
Anders Løbner-Olesen
Pierre Léopold
Céline Lévesque
Maris Laan
Pierrick Labbé
Michel Labouesse
Soni Lacefield
Andrea Ladd
Christine Ladd-Acosta
Raj Ladher
Peter Ladurner
Michael Laessig
Thierry Lagrange
Thomas Lahaye
Robert Lahue
Zhi-Chun Lai
Marikki Laiho
Nigel Laing
Cathleen Lake
Dolores Lamb
Sarah Lambert
Gregory Lang
Bodo Lange
Thomas Langer
Oscar Lao
Laurent Laplaze
Rebecca Laposa
Tuuli Lappalainen
Luis Larrondo
Paul Lasko
Wyndham Lathem
Nelson Lau
Michael Laub
Vincent Laudet
Adam Lauring
Martin Lavin
Laura Lavine
Matthew Law
Mara Lawniczak
Nathan Lawson
Iosif Lazaridis
Brian Lazzaro
Arnaud Le Rouzic
Rod Lea
David Leach
Matthew Lebo
Seung-Jae Lee
Cheng-Yu Lee
Siu Sylvia Lee
Siu Lee
Hyoung-gon Lee
Grace Lee
Jong-Min Lee
Hangnoh Lee
Vincent Lee
Sang Lee
Changhan Lee
Michael Lee
Byungwook Lee
Tosso Leeb

Peter Leegwater
Jeffrey Leek
Susan Lees-Miller
Michael Leffak
Ellen Leffler
Gaëlle Legube
Ruth Lehmann
Ordan Lehmann
Christian Lehner
Ben Lehner
Birgit Leitinger
Sylvain Lemeille
Bernardo Lemos
Georg Lenz
Virpi Leppa
Johanna Leppälä
Maria Leptin
Stephen Leslie
Sylvain Lesne
Marci Lesperance
Christopher Lessard
Noelle L'Etoile
Guillaume Lettre
Petra Levin
Edward Levine
Joel Levine
Gil Levkowitz
Daniel Lew
Zachary Lewis
Brian A Lewis
Peter Lewis
Xiajun Li
Wen-Qing Li
Xin Li
Qi-Jing Li
Sheng Li
Bingshan Li
Jun Li
Chao Li
Yun Li
Jigang Li
Chuanyou Li
Bo Li
Tiansen Li
Xiao-Jiang Li
Mingyao Li
Chris Li
Jianming Li
Xiaojinag Li
Hong-Sheng Li
Shaoguang Li
Liming Liang
Giordano Liberi
Domenico Libri
Alexander Lichius
Jonathan Licht
Juergen Liebig
Susan Liebman
Graham Lieschke
Roland Lill
Daniel Lim
Kah-Leong Lim
Han Lim
Su-Ju Lin
Xiaorong Lin
Rongcheng Lin
Rueyling Lin
Jing-Jer Lin
Chentao Lin
Amy Linabery
Paul Lindahl
Cecilia Lindgren
Gabriella Lindgren
Charlotte Ling
Jiqiang Ling
Joachim Lingner
Leonard Lipovich
Zachary Lippman
Damon Lisch
Ryan Lister
James Lister
Jennifer Listgarten
Gordon Lithgow
Tom Little
Nianjun Liu
Chunyu Liu
Edison Liu
Paul Liu
Jun Liu
Xuezhong Liu
Chun-Ming Liu
Dajiang Liu
Jimmy Liu
Gabriel Livera
Mats Ljungman
Bertrand Llorente
Alan Lloyd
Joyce A. Lloyd
Roger Sheng-Chieh Lo
Mary Lobo
Camille Lobry
Joseph Locker
Lawrence Loeb
Edward Loechler
Stacie Loftus
Malcolm Logan
Hannes Lohi
Timothy Lohman
Kirk Lohmueller
Konrad Lohse

Fanxin Long
Manyuan Long
Jeffrey C. Long
Greg Longmore
Michelle Longworth
Joseph Loparo
Javier Lopez
Jose Lopez-Ribot
Aaron Lorenz
Oscar Lorenzo
Christian Lorson
Ana Losada
Joseph Loscalzo
K. E. Lotterhos
Joseph LoTurco
Xiang-Yang Lou
Edward Louis
Kate Loveland
Michael Lovett
Susan Lovett
Christopher Lowe
Congming Lu
Qing Lu
Xiaowei Lu
Ting Lu
Sheng Luan
Francesca Luca
Michael Ludwig
Ulrich Luhmann
Brian Luke
Lawrence Lum
Derek Lundberg
Vicki Lundblad
Erik Lundquist
David Lunt
Gerton Lunter
Mathieu Lupien
Aldons Lusis
Arthur Lustig
Joe Lutkenhaus
Lucio Luzzatto
David Lydall
Frank Lyko
Jeremy Lynch
Vincent Lynch
Gholson Lyon
Leslie Lyons
Jeffery Lysiak
Katrina Lythgoe
Yi Ma
Hong Ma
Li Ma
Dengke Ma
Li Jun Ma
Wenbo Ma
Richard Maas
John Mably
David MacAlpine
Paul Macdonald
Clinton MacDonald
Stuart Macdonald
Marcy MacDonald
Todd Macfarlan
Sara Macias
Alyson MacInnes
Ian Mackay
Trudy Mackay
Susan Mackem
Amy MacQueen
Frank Madeo
Morris Maduro
Christa Maes
Dixie Mager
Keith Maggert
Bridget Maher
Shamoni Maheshwari
Moe Mahjoub
Volker Mai
Berenike Maier
Thomas Mailund
William Mair
Alexis Maizel
Jacek Majewski
Ho Yi Mak
Svetlana Makovets
Pal Maliga
Harmit Malik
Anna Malkova
Antonello Mallamaci
Allison Mallory
Anders Malmendal
Julin Maloof
Gail Mandel
Riccardo Manganelli
Valentina Mangano
Arya Mani
Ani Manichaikul
Richard Mann
Mattias Mannervik
Louis Mansky
Richard Maraia
Gabriel Marais
Richard Marais
Costas Maranas
Joseph Maranville
William Margolin
Urko Marigorta
Jean-Christophe Marine
Federica Marini
Anna Maria Marini

John Marioni
Therese Markow
Michael Marks
Robert Martienssen
Adam Martin
James Martin
Olivier Martin
Francis Martin
Mark Martindale
Juan Pedro Martinez-Barbera
Juan Martinez-Cruzado
Enrique Martinez-Perez
Emmanuelle Martini
Jean-Claude Martinou
Nelson Martins
William Marzluff
Nicholas Masca
Thorsten Mascher
Joanna Masel
Daniel Masison
Dmitri Maslov
Christopher Mason
Catherine Masson-Boivin
A. Gregory Matera
Christopher Mathew
Christopher Mathews
Iain Mathieson
Ivan Matic
Noriyuki Matsuda
Ichiro Matsumoto
John Mattick
Marjori Matzke
Martin Matzuk
Cedric Maurange
Matthew Maurano
Celia May
Thibault Mayor
Roberto Mayor
Didier Mazel
Heidi McBride
Kevin McBride
Brian McCabe
Andrew McCallion
John McCarrey
Steven McCarroll
John McCarthy
David McClay
Dannel McCollum
Richard McCulloch
Bruce McDonald
Donald McDonnell
Michael McEachern
Ken McElreavey
James McGhee
William McGinnis
Trudy McKay
Daniel McKay
Bruce McKee
Paul McKeigue
Kim McKim
Brett McKinney
Peter McKinnon
Aoife McMahon
Joel McManus
David McMillen
Michael McMurray
Elizabeth McNally
Allan McRae
Gil McVean
Mitch McVey
Graham McVicker
Vivien Measday
Marcel Mechali
Sarah Medland
Juan Medrano
Jack Meeks
Heather Mefford
Colin Meiklejohn
Friedhelm Meinhardt
Miriam Meisler
Gunter Meister
Cathryn Mellersh
Karyn Meltz Steinberg
Cathy Mendelsohn
Anna Menini
Horacio Merchant-Larios
Raphaël Mercier
Karine Merienne
Houra Merrikh
Bradley Merrill
Diane Merry
Anne Messer
Philipp Messer
Ludwine Messiaen
Markus Metzler
Theodorus Meuwissen
Dirk Meyer
Justin Meyer
Blake Meyers
Stephen Meyn
Christine Mezard
Joseph Miano
Matthew Michael
Pawel Michalak
Bénédicte Michel
Jan Michiels
Gracjan Michlewski
Tâm Mignot
Dusanka Milenkovic
Michael Miller

Dana Miller
Kyle Miller
Wolfgang Miller
Craig Miller
Yury Miller
David Miller III
Kevin Mills
Aleksandar Milosavljevic
Chieka Minakuchi
Michal Minczuk
Maria Caterina Mione
Olivo Miotto
Mario Mirisola
Sergei Mirkin
Christen Mirth
Caterina Missero
Charlotte Mistretta
David Mitchell
Phil Mitchell
Ortrun Mittelsten Scheid
Gerhard Mittler
Masaaki Miyamoto
Tim Miyashiro
Danesh Moazed
Harry Mobley
Naoki Mochizuki
Kazufumi Mochizuki
Cecilia Moens
Axel Mogk
Karen Mohlke
Isabella Moll
Barbara Moller
Peter Mombaerts
Fernando Monje-Casas
Raymond Monnat
Jacques Montagne
Elena Monte
Freddy Monteiro
Antónia Monteiro
Craig Montell
Stephen Montgomery
Kristi Montooth
Sean Moore
Claire Moore
Priya Moorjani
Stephen Moose
Carlos Moraes
John Moran
Charles Moran
Kevin Morano
Robert Morell
Roy Morello
Kelley Moremen
Celine Morey
Gareth Morgan
Alan Morgan
Peter Morrell
Andrew Morris
Glenn Morris
Joachim Morschhäuser
Randall Morse
Geert Mortier
Ettore Mosca
Michele Moschetta
Matthew Moscou
Alan Moses
Rebecca Mosher
Larry Moss
Elisa Motori
Richard Mott
Arnaud Mourier
Ahmed Moustafa
Richard Moxon
Gary Muehlbauer
Ralf Mueller
Juerg Mueller
Louis Muglia
M. Shahid Mukhtar
Albert Mulenga
Stefan Muljo
Charles Mullighan
Mary Mullins
James Mulloy
Kasper Munch
John Mundy
Nicholas Mundy
Christopher Mungall
Carol Munro
Mary Munson
Alysson Muotri
Shunichi Murakami
Maureen Murphy
Coleen Murphy
Peter Murray
Richard Murray
Kiran Musunuru
Marco Muzi-Falconi
Chad Myers
Lawrence Myers
Peter Myler
Ulrich Nübel
Kentaro Nabeshima
Felix Naef
Emi Nagoshi
Laszlo Nagy
Yusaku Nakabeppu
Keiji Nakajima
Toru Nakamura
Yuki Nakamura
Michiko Nakano

Jun-Ichi Nakayama
Mike Nalls
Keiichi Namba
Olivier Namy
Geeta Narlikar
Steven Narod
Iñigo Narvaiza
Dick Nassel
Sheila Nathan
Arcadi Navarro
Pablo Navarro
Béatrice Nawrocki-Raby
Matthew Neale
Richard Neher
Joel Neilson
Maurine Neiman
Pete Nelson
Heather Nelson
William Nelson
Christian Neri
Scott Ness
Daniel Nettersheim
Thomas Neufeld
Karla Neugebauer
Paul Newcombe
Ashley Ng
Pauline Ng
Robert Nibbs
Jennifer Nichols
Jac A. Nickoloff
Simone Niclou
Alain Nicolas
Stefania Nicoli
Michael Niederweis
Conrad Nieduszynski
Kirsten Nielsen
William Nierman
Carien Niessen
Philipp Niethammer
Yoshihito Niimura
H. Nijhout
Zachary Nimchuck
Marc Nishimura
Deng-Ke Niu
Ryusuke Niwa
Marcelo Nobrega
Justin Nodwell
Eishi Noguchi
Yoo-Sun Noh
Michael Nonet
Francis Noonan
Mohamed Noor
Alexander Nord
Magnus Nordborg
Patrik Nosil
John Novembre
Minou Nowrousian
Noa Noy
Evgeny Nudler
Constance Nugent
Thomas Nystrom
Andrzej Ożyhar
John Oakeshott
Berl Oakley
Darren Obbard
Anita Oberbauer
Thomas O'Brien
Moira O'Bryan
Donal O'Carroll
Vincent O'Connor
Duncan Odom
Liza O'Donnell
Janis O'Donnell
Michael O'Donovan
Karen Oegema
Patrick O'Farrell
Joe Ogas
Yuya Ogawa
Stacey Ogden
Marco Oggioni
Edwin Oh
Kazuyuki Ohbo
Hiroyuki Ohkura
Kevin Ohlemiller
Benjamin Ohlstein
Misato Ohtani
Yukinori Okada
Giles Oldroyd
Dominik Oliver
Jeanine Olsen
James Olzmann
Rachel O'Neill
Daria Onichtchouk
Ariel Orellana
Moshe Oren
David Ornitz
Dennis O'Rourke
Terry Orr-Weaver
Henrik Oster
Mike Ostrowski
Jacintha O'Sullivan
Paul O'Toole
George O'Toole
Martin Ott
Marit Otterlei
Ruth Ottman
Edgar Otto
Sarah Otto
Guangshuo Ou
Ivan Ovcharenko

Stefanie Pöggeler
Brigitte Pützer
Badri Padhukasahasram
Grier Page
David Page
Andrea Page-McCaw
Athma Pai
Javier Palatnik
Ravi Palavinelu
Alexander Palazzo
Leo Pallanck
Mark Pallen
Abraham Palmer
Tom Palmer
Colin Palmer
Aarno Palotie
Arnar Palsson
Mala Pande
Udai Pandey
Vlad Panin
Michael Pankratz
V. Pankratz
Anne Paoletti
Joseph Papamatheakis
Manolis Papamichos-Chronakis
Silvia Paracchini
Fernando Pardo-Manuel de Villena
Mary-Lou Pardue
Peter Pare
David Parichy
Yoonseong Park
Roy Parker
Martin Parniske
Renato Paro
Ze'ev Paroush
Geraint Parry
John Parsch
Matthew Parsek
Michael Parsons
Mark Parthun
Linda Partridge
Philippe Pasero
Amy Pasquinelli
Geraldo Passos
Joao Passos
Tomi Pastinen
Smita Patel
Lavinia Paternoster
Andrew Paterson
Nikolaos Patsopoulos
Andrea Pauli
Tanya Paull
Ralf Paus
William Pavan
Youri Pavlov
John Pawelek
Wojciech Pawlowski
Gregory Pazour
Warren Pear
Christopher Pearson
Lotte Pedersen
Itsik Pe'er
Catherine Peichel
Mark Peifer
Jason Peiffer
Francisco Pelegri
Miguel Penalva
Alberto Pendas
Pleuni Pennings
Marta Perego
Filipe Pereira
Andrew Perkins
George Perry
John Perry
Michael Perry
Sharyn Perry
Christoph Peterhänsel
Antoine Peters
Jason Peters
Christian Petersen
Svend Petersen-Mahrt
David Peterson
Thomas Peterson
Craig Peterson
Thomas Petes
Slave Petrovski
Nitin Phadnis
Paul Pharoah
Megan Phifer-Rixey
Patrick Phillips
Eric Phizicky
Manuel Piñeiro
Stefano Piccolo
Franck Pichaud
Joseph Pickrell
Alison Pidoux
Eric Pierce
Jonathan Pierce-Shimomura
Francesc Piferrer
Francesca Pignoni
Lori Pile
Henriette Pilegaard
Ramesh Pillai
Marc Pilon
John Pimanda
Zachary Pincus
Roger Pique-Regi
Chrysoula Pitsouli
Jeffrey Pleiss
Scott Pletcher

Robert Plomin
Andrew Pocklington
Roger Pocock
Benjamin Podbilewicz
Frank Poelwijk
R. Poethig
Martin Pollak
Daniel Pollard
Thomas D. Pollard
Ana Pombo
Andrew Pomiankowski
Ricardo Pong-Wong
John Pool
Art Poon
Maria Poptsova
Mary Porter
Douglas Portman
James Posakony
Kenneth Poss
Danielle Posthuma
Gino Poulin
Olivier Pourquie
Jeffrey Powell
Juan Poyatos
Félix Prado
Supriya Prasanth
Marcela Preininger
Thomas Preiss
Jessica Prenni
Daven Presgraves
Alice Pressman
Stephen Price
Clive Price
Carolyn Price
Alkes Price
Jeffrey Price
James Priess
Victoria Prince
Daniel Promislow
Nick Proudfoot
William Pu
M. Graciela Pucciarelli
Jennifer Puck
Rosa Puertollano
Pere Puigserver
Nathalie Pujol
Patricia Pukkila
Shaun Purcell
Zachary Pursell
Michael Purugganan
Harald Putzer
Joshua Puzey
Ling Qi
Ji Qi
Wenfeng Qian
Hong Qiao
Feng Qiao
Li-Jia Qu
Lynne Quarmby
Christine Queitsch
Ethel Queralt
Tom Quertermous
David Quigley
Christopher Quince
Lluis Quintana-Murci
Arshed Quyyumi
Marius Rösti
Oliver Rackham
Alvaro Rada-Iglesias
Daniel Rader
Pablo Radicella
Jerry Radich
Lori Raetzman
M Raghuraman
David Raible
Alexander Raikhel
Towfique Raj
Vardhman Rakyan
Peter Ralph
Sohini Ramachandran
Kumaran Ramamurthi
Baranidharan Raman
Maria Ramirez
David Rand
Mahendra Rao
Christopher Rao
Allison Rattray
Frank Rauch
Tommer Ravid
John Rawls
Anandasankar Ray
Soumya Raychaudhuri
Laurie Read
Mark Rebeiz
Mario Recker
Peter Reddien
Timothy Reddy
Maria Redondo
Roger Reeves
Birgitte Regenberg
Jean-Marc Reichhart
Martin Reijns
Muredach Reilly
Alan Rein
David Reiner
Thomas Reinheckel
Hans Reinke
Olaf Reiss
Lawrence Reiter
Ofer Reizes

Stephen Renshaw
Martijn Rep
Elizabeth Repasky
Michael Resnick
Seung Rhee
Nick Rhind
Ulf Ribacke
Ezio Ricca
Anthony Ricci
Christina Richards
Thomas Richards
Christine Richardson
Helena Richardson
Ann Richmond
Klaus Richter
Christopher Ridout
Michelle Riehle
Loren Rieseberg
Arne Rietsch
Jason Rihel
Yasuko Rikihisa
Brigit Riley
Bruce Riley
Christoph Ringli
Leonie Ringrose
Niels Ringstad
Jochen Rink
Samuli Ripatti
Jürgen Ripperger
Michael Ristow
Marylyn Ritchie
Manuel Rivas
Silke Robatzek
Francois Robert
Charles Roberts
Peter Robinson
D. Ashley Robinson
Paul Robson
Aldo Roccaro
Eduardo Rocha
Christian Rocheleau
Jason Rock
Matthew Rockman
Claudina Rodrigues-Pousada
Enrique Rodriguez-Boulan
Fabrice Roegiers
Guus Roeselers
Stephen Rogers
Ignasi Roig
Jean-Yves Roignant
Darin Rokyta
Gregg Roman
Luisa Romao
David Ron
Ze'ev Ronai
Lars Ronnegard
Stephane Ronsseray
Dennis Roop
Randall Roper
Evan Rosen
Vicki Rosen
Jeffrey Rosen
Susan Rosenberg
Miriam Rosenberg
Georg Rosenberger
Emanuel Rosonina
Eric Ross
Joseph Ross
Jeffrey Ross-Ibarra
Elizabeth Rossin
John Roth
Lawrence Rothblum
Adrian Rothenfluh
Joseph Rothnagel
Veerle Rottiers
Claire Rougeulle
Michael Rout
François Rouyer
Pierre Rouzé
David Rowitch
Scott Roy
Peter Roy
Richard Roy
Joshua Rubin
David Rudner
Michael Rudnicki
Christian Rudolph
Davide Ruggero
Natividad Ruiz
Eduardo Ruiz-Pesini
Laura Rusche
Paul Russell
Antonio Russo
Tor Erik Rusten
Julian Rutherford
Tom Rutkowski
Ilya Ruvinsky
Gary Ruvkun
Robert Ryan
Ivan Rychlik
Sean Ryder
Hyung Don Ryoo
Dieter Söll
Ann Saada
Matthew Sachs
Takashi Sado
Paul Saftig
Yumiko Saga
Alvaro Sagasti
Julien Sage

Isabelle Sagot
Yusuke Saijo
Hiroyuki Sakurai
Nina Salama
Julian Sale
Iris Salecker
David Salomon
Paul Salvaterra
Helen Salz
Christos Samakovlis
Kaitlin Samocha
Maurilio Sampaolesi
David Samuels
Albin Sandelin
Maria Sandkvist
Dan Sanes
Tzu-Kang Sang
Kaustuv Sanyal
Juan Sanz-Ezquerro
Tomasz Sarnowski
Vittorio Sartorelli
Makoto Sato
Tatjana Sauka-Spengler
Simon Saule
Harald Saumweber
Herbert Sauro
Sharon Savage
Elizabeth Savelkoul
Nigel Savery
Outi Savolainen
Hitoshi Sawa
Ritwick Sawarkar
Stephen Sawcer
Peter Scacheri
Enrico Scarpella
Frieder Schöck
Beat Schaefer
Patrick Schafer
G. Schaller
Tim Schedl
Paul Schedl
Dirk-Jan Scheffers
Eyal Schejter
Annette Schenck
Aloys Schepers
Bernhard Schermer
Elmar Schiebel
Alexander Schier
Mario Schiffer
Philipp Schiffer
Joshua Schiffman
Carl Schildkraut
Thomas Schilling
John Schimenti
Ernestina Schipani
Christian Schlötterer
Todd Schlenke
Peter Schloegelhofer
Patrick Schloss
Andreas Schlosser
Dolph Schluter
Marion Schmidt
Martin Schmidt
Thomas Schmidt
Paul Schmidt
Urs Schmidt-Ott
Manfred Schmitt
Robert Schmitz
Sheila Schmutz
Korbinian Schneeberger
David Schneider
Guenter Schneider
Karin Schnetz
Arp Schnittger
Frank Schnorrer
Jeffrey Schoenebeck
Eric A. Schon
Gunnar schotta
Daniel Schrider
Dirk Schubeler
Markus Schuelke
Erin Schuetz
Johannes Schulte
Patricia Schulte
Michael Schultz
Cordula Schulz
Jill Schumacher
Heribert Schunkert
Tanja Schwander
François Schweisguth
Beate Schwer
James Schwob
Luca Scorrano
Kristin Scott
Donald Scott
Barry Scott
William Scott
Paul Scotting
Paola Sebastiani
John Sedivy
Marisa Segal
Mark Seielstad
H. Seifert
Jeff Sekelsky
Vimal Selvaraj
Elena Semina
Saunak Sen
Piali Sengupta
Cathal Seoighe
Bertrand Seraphin

Tricia Serio
Florenci Serras
Susan Service
Bertrand Servin
Ed Seto
Claudio Sette
Andrey Shabalin
Nirao Shah
Shai Shaham
Catherine Shanahan
Beth Shapiro
Cynthia Sharma
Itai Sharon
Richard Sharp
Andrew Sharp
Richard Sharpe
Joseph Shaw
Paul Shaw
Polina Shcherbakova
Val Sheffield
Kang Shen
Xia Shen
Jay Shendure
Sanjay Shete
Frank Shewmaker
Yongshen Shi
Jianxin Shi
Takehiko Shibata
Darryl Shibata
Jung-Bum Shin
Katsuhiko Shirahige
Shin-Han Shiu
Robert Shmookler Reis
Lindsay Shopland
David Shore
James Shorter
Eric Shoubridge
Sadeep Shrestha
Daniel Shriner
Mark Shriver
Leah Shriver
Stanislav Shvartsman
Elaine Sia
Gabriele Siciliano
Julia Sidorova
Ellen Sidransky
Derek Sieburth
Tim Siegel
Deborah Siegele
Stephan Sigrist
Olin Silander
Matt Silver
Andrew Silver
Giora Simchen
Henner Simianer
Rebecca Simmons
Lyle Simmons
Christipher Simmons
Marie-Noelle Simon
Martine Simonelig
Yuval Simons
Kai Simons
Anne Simonsen
Andrew Sinclair
Charles Sing
Nadia Singh
Saurabh Sinha
Neelima Sinha
Himanshu Sinha
Haruhiko Siomi
Manuela Sironi
Bjørn Steen Skålhegg
Robert Skibbens
Theodorus Sklaviadis
Pontus Skoglund
Christopher Slape
Jon Slate
Steven Slater
James Slauch
Barry Sleckman
Daniel Sloan
Jason Slot
R Slotkin
Michel Slotman
Guy Smagghe
Ian Small
Craig Smibert
Marten Smidt
Alicia Smith
Jeffrey Smith
Lisa Smith
Rachel Smith-Bolton
Marcus Smolka
Emilie Snell-Rood
Michael Snyder
Darren Soanes
Yoav Soen
Maria Soengas
Jordi Solana
Lilianna Solnica-Krezel
Paul Soloway
Nahum Sonenberg
Daniel Sonenshine
Giltae Song
Kevin Sonnemann
Rashmi Sood
Peter Sorensen
Melissa Southey
Christian Speck
Doug Speed

Johannes Spelbrink
Thomas Spencer
Silke Sperling
Salvatore Spicuglia
Charles Spillane
Allan Spradling
Simon Sprecher
Nathan Springer
David Sprinzak
Duncan Sproul
Supriya Srinivasan
Jagan Srinivasan
Eli Stahl
Dorothee staiger
Stefan Stamm
Ralf Stanewsky
Peter Stanton
Ann Stapleton
Daniel Starczynowski
Michael Stark
Jeremy Stark
Doekele Stavenga
Tim Stearns
Camille Steber
Karen Steel
Oliver Stegle
Gero Steinberg
Eirikur Steingrimsson
Lars Steinmetz
Olaf Stemmann
Marcus Stensmyr
Anna Stepanova
David Stern
Paul Sternberg
Tyler Stevenson
James Stewart
A. Francis Stewart
Stefano Stifani
Bruce Stillman
Ann Stock
Hugo Stocker
John Storey
Gary Stormo
Jay Storz
Gisela Storz
Travis Stracker
Aaron Straight
Paul Straight
George Stratigopoulos
Tobias Straub
Joseph Strauss
Sam Strom
Lisa Strug
Kevin Struhl
Ken Stuart
Lisa Stubbs
Eva Stukenbrock
Jorg Stulke
Richard Sturm
Chih-Ying Su
Tin Tin Su
Susan Suarez
Shankar Subramaniam
Garret Suen
Katsunori Sugimoto
Takuya Sugiyama
Greg Suh
Karsten Suhre
Jose Suja
Beth Sullivan
William Sullivan
Zhaoxia Sun
Yi Henry Sun
Lei Sun
Yi Sun
Meera Sundaram
Patrick Sung
Z. Renee Sung
Mark Sussman
Mark Sutton
Jesper Svejstrup
Michael Swarbrick
James Swenberg
Elizabeth Swisher
Anne Sylvester
Lorraine Symington
V. Symonds
Ann-Christine Syvänen
Roman Szabo
Jan-Willem Taanman
Dylan Taatjes
Carter Takacs
Ryosuke Takahashi
Minoru Takata
Shunichi Takeda
Yusuke Takehana
Frank Takken
Shohei Takuno
Paul Talbert
John Tamkun
Guiliang Tang
Robert Tanguay
Toshiyasu Taniguchi
Stefan Taubert
Sean Taverna
Martin Taylor
Hugh Taylor
Guy Tear
Martin Teichmann
Maria Teresa Teixeira

Luis Teixeira
Aurelio Teleman
Amalio Telenti
Adam Telerman
Liesbet Temmerman
Alan Templeton
Derk ten Berge
Peter ten Dijke
Sheng Teng
Shu-Chun Teng
Jason Tennessen
Yik-Ying Teo
Akihisa Terakita
Sotirios Tetradis
Maria Teves
Martin Thanbichler
Mukund Thattai
Thomas Theil
Ulrich Theopold
William Theurkauf
Johan Thevelein
Bernard Thienpont
Christopher Thomas
David Thomas
Tim Thomas
Wellems Thomas
Rachael Thomas
Sebastien Thomine
Charles Thompson
Barry Thompson
Dawn Thompson
Genevieve Thon
Stefan Thor
David Thorburn
Kevin Thornton
Joseph Thornton
Timothy Thornton
Unnur Thorsteinsdottir
David Threadgill
Carl Thummel
Ryan Thummel
Thorsten Thye
Randal Tibbetts
Peter Tiffin
Vincent Timmerman
Marja Timmermans
H.Th.Marc Timmers
Andrew Timms
Laurence Tiret
Sarah Tishkoff
Heidi Tissenbaum
Rune Tofgard
Pavel Tomancak
Vittorio Tomasi
Lubomir Tomaska
Kozo Tomita
Yoshinori Tomoyasu
Stephen Tonsor
Tina Tootle
Jorma Toppari
Laszlo Tora
Silvia Tornaletti
Esteban Toro
Maria-Elena Torres-Padilla
Jorg Tost
Attila Toth
John Tower
Paul Trainor
Gregory Tranah
Jacquetta Trasler
Ana Traven
Michael Travisano
Bryan Traynor
Jessica Treisman
David Tremethick
Jeffrey Trent
Susannah Tringe
Emily Troemel
Didier Trono
Heather True-Krob
Marco Trujillo
Gosia Trynka
Yuk-Ching Tse-Dinh
Bryan Tsou
Stephen Kwok-Wing Tsui
Shinobu Tsuzuki
Priscilla Tucker
Paul Tudzynski
Mick Tuite
Taru Tukiainen
Franziska Turck
Leslie Turner
Paul Turner
Thomas Turner
Bryan Turner
Simon Turner
James Turner
Jens Tyedmers
Alexander Tzagoloff
Marius Ueffing
Helle Ulrich
Robert Unckless
Dustin Updike
Alexander Urban
Raul Urrutia
Michele Vacca
Saba Valadkhan
Leos Valasek
Juan Valcarcel
Raphael Valdivia

Eivind Valen
Alfonso Valencia
Philip Van Damme
Martijn van de Bunt
Esther van de Vosse
Wynand van der Goes van Naters
Ransome van der Hoeven
Jan van der Meer
Marjan van der Woude
Patrick Van Dijck
Karine Van Doninck
Mark Van Doren
Dik van Gent
Ambro van Hoof
Jeremy Van Raamsdonk
Harry van Steeg
Pieter Van Vlierberghe
Vicki Vance
Jozef Vanden Broeck
Carin Vanderpool
Juan Vaquerizas
Soory Varambally
Michael Vasil
Daniel Vasiliauskas
Lidia Vasiljeva
Karen Vasquez
Hervé Vaucheret
Jan-Willem Veening
Xavier Vekemans
Julian Venables
Andre Verdel
Eric Verdin
Paul Verdu
Louis Vermeulen
Teva Vernoux
Rogier Versteeg
Patrik Verstreken
Michel Vervoort
Jean-Baptiste Veyrieras
Ana Viñuela
Marc Vidal
Miguel Vidal
Alexandre Vieira
Cristina Vieira
Jean-Philippe Vielle-Calzada
Jose Vilar
Andreas Vilcinskas
Bjarni Vilhjalmsson
Johanna Vilkki
Francesc Villarroya
Anna Vinkhuyzen
Neus Visa
Rosella Visintin
David Viskochil
Theo J. Visser
Jerry Vockley
Christine Vogel
Benjamin Voight
Renate Voit
Talila Volk
Pelin Cayirlioglu Volkan
Erik Vollbrecht
Tobias von der Haar
Klaus Von der Mark
Marieke von Lindern
Andrea Vortkamp
Rome Voulhoux
Masaaki Wachi
Andreas Wachter
Scott Waddell
Nicola Waddell
Günter Wagner
Fergal Waldron
Marian Walhout
Harkamal Walia
David Walker
Daniel Wall
Jason Wallace
Chris Wallace
John Wallingford
Kyle Walsh
Martin Walsh
Colum Walsh
Timothy Walsh
Jörn Walter
Ronald Walter
Lucas Waltzer
Jianmin Wan
Tao Wang
Greg Wang
Dong Wang
Wei Wang
Yue Wang
Xinnan Wang
Zhe Wang
Ya Wang
Baolin Wang
Hansong Wang
Biao Wang
Clay Wang
Zhiyong Wang
Haiyang Wang
Hao Wang
Guo-Liang Wang
Jue Wang
P. Jeremy Wang
Philine Wangemann
Kevin Wanner
Elizabeth Want
Fiona Wardle

Wesley Warren
Yoshinori Watanabe
Nicholas Waterfield
Chris Waters
Erica Watson
Jennifer Watts
Valerie Weaver
Michael Weber
Matthew Webster
Michael Weedon
Dan Weeks
David Weetman
Michael Wegner
Yau-Huei Wei
Detlef Weigel
Alan Weiner
Ted Weinert
Michael Weinfeld
Mitchell Weiss
Lauren Weiss
David Weiss
Daniel Weissman
Charles Weitz
Ronald Wek
Piri Welcsh
Paul Welling
Raymund Wellinger
GG Welstead
Michael Welte
Xiaoquan Wen
JIn-kun Wen
Hans-Guido Wendel
Jonathan Wendel
Jing-Ke Weng
Sabine Werner
John Werren
Joel Wertheim
Robert Wessells
Benedikt Westermann
Lynn Westphal
Kristi Wharton
Eleanor Wheeler
Vanessa Wheeler
Rachel Whitaker
Rob White
Kevin White
Ian White
Bradley White
Frank White
Robert White
Stefan White
Emma Whitelaw
Malcolm Whiteway
Tanya Whitfield
Michael Whitlock
Alexander Whitworth
Michael Whyte
Max Wicha
Reed Wickner
Philipp Wiemann
Bé Wieringa
Andrzej Wierzbicki
Eric Wieschaus
Cisca Wijmenga
Ellen Wijsman
Claus Wilke
Lynne Wilkens
Rob Willemsen
Cristen Willer
Trevor Williams
Sion Williams
Judith Willis
Hugh Willison
Michael Wilson
Geoffrey Wilson
James Wilson
David Wilson
Samuel Wilson
Daniel Wilson
Scott Wilson
James F. Wilson
Richard Wilson
Tim Wiltshire
Jeffrey Wilusz
Klaus Wimmers
Susan Winandy
Rebecca Wingert
Wade Winkler
Craig Winstanley
Fred Winston
Mary Wirtz
John Witte
Curt Wittenberg
Patricia Wittkopp
Ralph Witzgall
Joseph Witztum
Fred Wolf
Kenneth Wolfe
Debra Wolgemuth
Lee-Jun Wong
Alex Wong
Chloe C Y Wong
Tien Wong
Andrew Wood
Neil Woodford
Roger Woodgate
Naomi Wray
Anna Wredenberg
Carl Wu
Rongling Wu

C.-ting (Ting) Wu
Hen-ming Wu
Zhihao Wu
Qingyu Wu
Chung-I Wu
Joao Xavier
Shunyuan Xiao
Zhixin Xie
Anyong Xie
Daoxin Xie
Qi Xie
Liming Xiong
Tongda Xu
Yaji Xu
Jian Xu
Shuhua Xu
Shizhong Xu
Jianping Xu
Chaoyang Xue
Kazuhiro Yagita
Gen Yamada
Ayumu Yamamoto
Toshiyuki Yamamoto
Ai Yamamoto
Kunitoshi Yamanaka
Yukiko Yamashita
Masakazu Yamazaki
Hua Yan
Jian-Rong Yang
William Yang
Shuhua Yang
Guojun Yang
Wei Cai Yang
HongQuan Yang
Judith Yanowitz
Song Yao
Ayse Yarali
Zheng-Hua Ye
Yihong Ye
De Ye
Keqiang Ye
Chun Ye
Laising Yen
Giles Yeo
Laura Yerges-Armstrong
Rui Yi
Eda Yildirim
Fitnat Yildiz
Bayam Yilmaz
Viravuth Yin
Yanhai Yin
Jerry Yin
Gregory Yochum
John Yoder
Yao Yonggang
Kohki Yoshimoto
Kevin Young
Nevin Young
Heather Young
Jianming Yu
Hongtao Yu
Fengwei Yu
Bin Yu
Jae-Hyuk Yu
Feng Yue
Wyatt Yue
Hong-wa Yung
Katherine Yutzey
Viktor Zárský
Ksenija Zahradka
Noah Zaitlen
Virginia Zakian
Harold Zakon
Peter Zammit
Phillip Zamore
Peter Zaphiropoulos
David Zappulla
Kenneth Zaret
Arieh Zaritsky
David Zarkower
Daniela Zarnescu
Judith Zaugg
Ricardo Zayas
Ronai Ze'ev
Scott Zeitlin
Zhao-Bang Zeng
Lirong Zeng
Martin Zenker
Monique Zetka
Massimo Zeviani
Grace Zhai
Zhiwu Zhang
Yong Zhang
Wei Zhang
Yuelin Zhang
Yun Zhang
Ye Zhang
Jinghui Zhang
Xiuren zhang
Kang Zhang
Xiaolan Zhang
Y-H Percival Zhang
Zhibing zhang
Dekai Zhang
Jing Zhao
Zhong Zhao
Meixia Zhao
Li Zhao
Mei Zhen
Alexandra Zhernakova

Yi Zhong
Xuehua Zhong
Hua Zhong
Baohua Zhou
Bing Zhou
Binhua Zhou
Jiliang Zhou
Rui Zhou
Gangqiao Zhou
Mike Zhu
Xiaofeng Zhu
Jun Zhu
Jinsong Zhu
Xiaoyu Zhuo
Denise Zickler
Daniel Zilberman
Laurent Zimmerli
Richard Zimmermann
Robert Zimmermann
Kai Zinn
Elad Ziv
Huda Zoghbi
Sebastian Zollner
Joost Zomerdijk
Peter Zuber
Stephan Zuchner
Jian Zuo
Michael Zwick
